# Rainbow Trout Erythrocytes *ex vivo* Transfection With a DNA Vaccine Encoding VHSV Glycoprotein G Induces an Antiviral Immune Response

**DOI:** 10.3389/fimmu.2018.02477

**Published:** 2018-10-29

**Authors:** Sara Puente-Marin, Ivan Nombela, Veronica Chico, Sergio Ciordia, Maria Carmen Mena, Julio Coll, Luis Mercado, Maria Del Mar Ortega-Villaizan

**Affiliations:** ^1^Instituto de Biología Molecular y Celular, Universidad Miguel Hernández, Elche, Spain; ^2^Unidad de Proteómica, Centro Nacional de Biotecnología (CNB-CSIC), Madrid, Spain; ^3^Instituto Nacional de Investigaciones Agrarias, Biotecnología, Madrid, Spain; ^4^Instituto de Biología, Pontificia Universidad Católica de Valparaíso Valparaíso, Chile

**Keywords:** rainbow trout, erythrocytes, red blood cells, VHSV glycoprotein G, DNA vaccine, transcriptome, proteome, immune response

## Abstract

Fish red blood cells (RBCs), are integral in several biologic processes relevant to immunity, such as pathogen recognition, pathogen binding and clearance, and production of effector molecules and cytokines. So far, one of the best strategies to control and prevent viral diseases in aquaculture is DNA immunization. DNA vaccines (based on the rhabdoviral glycoprotein G [gpG] gene) have been shown to be effective against fish rhabdoviruses. However, more knowledge about the immune response triggered by DNA immunization is necessary to develop novel and more effective strategies. In this study, we investigated the role of fish RBCs in immune responses induced by DNA vaccines. We show for the first time that rainbow trout RBCs express gpG of viral hemorrhagic septicaemia virus (VHSV) (GVHSV) when transfected with the DNA vaccine *ex vivo* and modulate the expression of immune genes and proteins. Functional network analysis of transcriptome profiling of RBCs expressing GVHSV revealed changes in gene expression related to G-protein coupled receptor (GPCR)-downstream signaling, complement activation, and RAR related orphan receptor α (RORA). Proteomic profile functional network analysis of GVHSV-transfected RBCs revealed proteins involved in the detoxification of reactive oxygen species, interferon-stimulated gene 15 (ISG15) antiviral mechanisms, antigen presentation of exogenous peptides, and the proteasome. Conditioned medium of GVHSV-transfected RBCs conferred antiviral protection and induced *ifn1* and *mx* gene expression in RTG-2 cells infected with VHSV. In summary, rainbow trout nucleated RBCs could be actively participating in the regulation of the fish immune response to GVHSV DNA vaccine, and thus may represent a possible carrier cells for the development of new vaccine approaches.

## Introduction

The fish immune system is characterized by an active innate immune system that is of primary importance in combating infections (1). However, differences between the fish immune system and that of mammals complicate the extrapolation of knowledge, thus limiting our ability to control infectious diseases in fish. Piscine infectious diseases, especially viral infections, cause significant losses globally, which implies a negative impact on aquaculture industry. For that reason, efforts have been made to understand the fish antiviral immune response over the last few decades. A particular area of interest is the role that nucleated red blood cells (RBCs) play as immune cell mediators (2–4).

In contrast to mammalian RBCs, fish RBCs are nucleated and contain organelles in their cytoplasm (5). In addition, RBCs contain transcriptome machinery that plays an active role in the immune response against viral infections (2). The involvement of nucleated RBCs in the immune response has been demonstrated both *in vivo* and *in vitro*. Moreover, RBCs can act as phagocytes and antigen-presenting cells and release cytokine-like factors such as interferon gamma that could affect macrophage function (6). Nucleated RBCs also are able to develop specific responses to different pathogen-associated molecular patterns (PAMPs) (4) and can modulate leukocyte activity by producing soluble factors (4, 7). Recently, it has been demonstrated that fish RBCs are also involved in the immune response to viral infections (7–10).

Currently, the prevention of viral diseases is only possible through vaccination or immune stimulation. In aquaculture, one of the best strategies for controlling and preventing viral diseases is DNA immunization (11). This method triggers long-term protection against viral infections (11). However, more knowledge about the immune response triggered by DNA immunization is necessary in order to develop new and more effective viral prevention strategies. It is necessary to understand both the viral molecules involved in triggering the host immune responses (immunogenicity and antigenicity) and the viral-induced immune host responses in efforts to improve current and develop new vaccination strategies. In addition, new applications targeting immune cells are being developed to improve the immune response of DNA vaccines (12).

At the present time, only DNA vaccines based on the rhabdoviral glycoprotein G (gpG) gene have been found to be effective for all fish rhabdoviruses tested (13, 14). However, the immune mechanisms responsible for their efficacy remain largely unknown. Therefore, this topic is of special importance to supplement the knowledge of how DNA vaccines confer immune protection, especially given the recent revelations of the importance of RBCs in the piscine immune response.

In this study, we show for the first time that rainbow trout RBCs can express the gpG of VHSV (GVHSV) and modulate the expression of genes related to interferon, such as interferon-inducible myxovirus resistance gene (*mx*), interferon-inducible RNA-dependent protein kinase (*pkr*), and interferon-induced protein with tetratricopeptide repeats 5 (*ifit5*). Transcriptomic and proteomic profiles network analyses revealed genes and proteins involved in G-protein coupled receptor (GPCR)-downstream signaling, complement activation, RORA-activates gene expression, ISG15 antiviral mechanisms, and detoxification of reactive oxygen species. Further, conditioned medium (CM) of GVHSV-transfected RBCs conferred protection to RTG-2 cell line against VHSV infection. Our results lead us to suggest that RBCs are immune cell mediators that play an active role in GVHSV DNA vaccine immune stimulation. Therefore, RBCs could be considered promising target or carrier cells in the development of new vaccine approaches.

## Materials and methods

### Animals

Rainbow trout (*Oncorhynchus mykiss*) of ~7–10 cm were obtained from a VHSV-free commercial farm (PISZOLLA S.L., CIMBALLA FISH FARM, Zaragoza, Spain), and maintained at the University Miguel Hernandez (UMH) facilities at 14°C. Prior to experiments, fish were acclimatized to laboratory conditions for 2 weeks.

### Cell cultures

RBCs were obtained from the peripheral blood of fish sacrificed by overexposure to tricaine (tricaine methanesulfonate, Sigma-Aldrich, Madrid, Spain) (0.2 g/L). Peripheral blood was collected from the caudal vein using insulin syringes (NIPRO, Bridgewater, NJ, USA). RBCs were purified by two density gradient centrifugations (1,600 rpm, Ficoll 1.007; Lymphoprep, Reactiva, Sigma-Aldrich) as previously described (8). Purified RBCs were placed in RPMI-1640 medium (Dutch modification) (Gibco, Thermo Fisher Scientific Inc., Carlsbad, CA) supplemented with 10% gamma irradiated fetal bovine serum (FBS) (Cultek, Madrid, Spain), 1 mM pyruvate (Gibco), 2 mM L-glutamine (Gibco), 50 μg/mL gentamicin (Gibco), 2 μg/mL fungizone (Gibco), 100 U/mL penicillin (Sigma-Aldrich), and 100 μg/mL streptomycin (Sigma-Aldrich). The cells were cultured at 14°C.

The rainbow trout cell line RTG-2 (Rainbow Trout Gonad-2) was purchased from the American Type Culture Collection (ATCC 50643) and maintained at 21°C in MEM medium (Sigma-Aldrich) containing 10% FBS, 1 mM pyruvate, 2 mM L-glutamine, 50 μg/mL gentamicin, and 2 μg/mL fungizone. RTS11, a rainbow trout monocyte/macrophage-like cell line (donated by Dr. Niels Bols) (15) isolated from a spleen hematopoietic culture was maintained at 21°C in Leibovitz's medium (L-15) (Sigma-Aldrich) supplemented with 20% FBS, 1 mM pyruvate, 2 mM L-glutamine, 50 μg/mL gentamicin, and 2 μg/mL fungizone.

### Antibodies

Primary antibodies used in the manuscript included rabbit polyclonal antibody against Mx protein produced at the laboratory of Dr. Amparo Estepa (16, 17), and mouse polyclonal antibodies against IL1β (interleukin 1 beta) (18, 19), IL8 (interleukin 8) (20), and TNFα (tumor necrosis factor alpha) (21) produced at the laboratory of Dr. Luis Mercado. A mouse monoclonal 2C9 antibody produced at laboratory of Dr. Julio Coll against the N protein of VHSV was used for VHSV labeling (22). For GVHSV labeling, we used a mixed of anti-GVHSV monoclonal antibodies (MAbs) (C10, 3F1A2, and I16) (23) produced at Dr. Julio Coll's laboratory. Secondary antibodies used in these studies included anti-rabbit IgG CF^TM^ 647 and anti-mouse IgG CF^TM^ 647 (Sigma-Aldrich, Madrid, Spain) produced in goat.

### Plasmids

Plasmid pmTFP1 (Allele Biotechnology, ABP-FP-TCNCS), encoding the teal fluorescent protein 1 (mTFP1) (24), used as control plasmid, and pmTFP1GVHSV, encoding mTFP1 fused to the C-terminus of the membrane gpG of VHSV (GVHSV) (GenBank accession A10182.1), described previously (25), were used for transfection assays.

### Cell transfection assays

RBC transfection assays were performed by electroporation using the Neon™ Transfection System (Life Technologies, Thermo Fisher Scientific, Inc.) one day after Ficoll purification. For each electroporation reaction, we used 4 μg of plasmid construct (pmTFP1 or pmTFP1GVHSV plasmid) per 1 × 10^6^ cells resuspended in Buffer T (Neon™ Transfection System Kit, Life Technologies). RBCs were electroporated at 1600 V, 30 ms, and 1 pulse and incubated at 14°C for one to six days in RPMI 10% FBS.

The RTS11 cell line was transfected by electroporation with 4 μg of plasmid construct (pmTFP1 or pmTFP1GVHSV) per 1·10^6^ cells using the Neon™ Transfection System and resuspended in Buffer R (Neon™ Transfection System Kit). RTS11 was electroporated at 1,600 V, 30 ms, and 1 pulse and incubated at 21°C for one to six days in L-15 20% FBS.

### Transcriptome analysis of FACS single-cell sorted GVHSV-expressing RBCs

**Figure 1:**
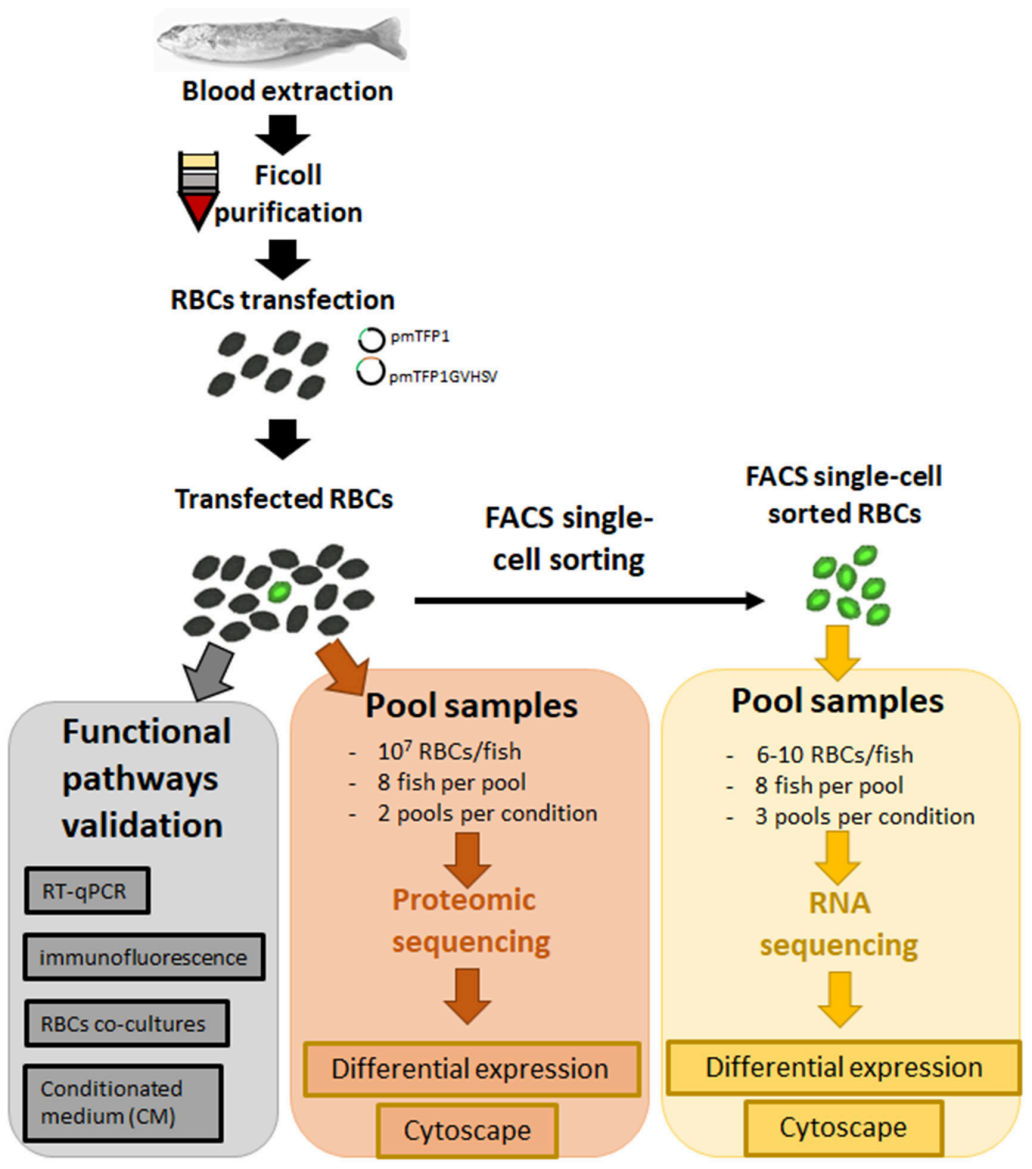
General workflow of experimental steps from sample collection to data analysis.

Ficoll-purified RBCs from 24 fish were transfected as described above with pmTFP1 or pmTFP1GVHSV (Figure 1). At six days post-transfection, TFP1- or GVHSV-expressing RBCs (6–10 cells per fish) were sorted by FACS single-cell sorting using the BD FACSJazz™ cell sorter (BD Biosciences, Madrid, Spain). FACS single-cell sorted RBCs were visualized in the IN Cell Analyzer 6000 Cell Imaging system (GE Healthcare, Little Chalfont, UK) (Figure S1). Each sample was resuspended in 9.5 μL of 10 × lysis buffer (Clontech, Takara Bio, Mountain View, CA, USA) and 0.5 μL of RNase inhibitor (Invitrogen, ThermoFisher Scientific, Waltham, MA, USA). Twenty-four fish samples were grouped in three pools of eight individuals for each condition (pmTFP1 or pmTFP1GVHSV) (Figure 1) and preserved at −80°C until cDNA library construction. Then, cDNA was directly produced from pooled lysed cells using SMART-Seq v4 Ultra Low Input RNA Kit (Clontech, Takara Bio) (26). Sequence reads are available at SRA-NCBI, SRA-NCBI Accession SRP133501. RNA-Seq library preparation and sequencing were carried out by STABVida Lda (Caparica, Portugal).

### Proteome analysis of transfected RBCs

Ficoll-purified RBCs from 16 fish were transfected as described above with pmTFP1 or pmTFP1GVHSV (Figure 1). At six days post-transfection, RBCs were pelletized by centrifugation (1,600 rpm), the supernatant was removed, and the cell pellet was washed three times with PBS, digested, and cleaned-up/desalted as previously described (26). Samples were pooled in two pools of eight individuals for each condition (pmTFP1 or pmTFP1GVHSV) (Figure 1). Then, samples were subjected to liquid chromatography and mass spectrometry analysis (LC-MS) as previously described (26), except that High pH Reversed-phase Peptide Fractionation Kit (Pierce, Thermo Fisher Scientific Inc.) was used for pH reversed-phase peptide fractionation, and four peptide fractions were collected. Progenesis QI v4.0 (Nonlinear Dynamics, Newcastle, UK) was used to analyze differential protein expression according to the “between-subject design.” Log_2_ peptide ratios followed a normal distribution that was fitted using least squares regression. Mean and standard deviation values derived from the Gaussian fit were used to estimate *P*-values and false discovery rates (FDR) at a quantitation level. The confidence interval for protein identification was set to ≥95% (*P*-value ≤ 0.05), and only peptides with an individual ion score above the 1% FDR threshold were considered to be correctly identified. Only proteins having at least two peptide spectrum matches (PSMs) were considered in the quantitation.

### Pathway enrichment analysis

Differentially expressed genes (DEGs) and differentially expressed proteins (DEPs) pathway enrichment analysis were performed using ClueGO (27) CluePedia (28), and Cytoscape (29). The GO Immune System Process, Reactome Pathway, and Reactome Reactions databases were used. A *P*-value ≤ 0.05 and Kappa score of 0.4 were used as threshold values. Genes and proteins were identified by sequence homology with *Homo sapiens* using Blast2GO version 4.1.9 Gotz (30).

### RTG-2 cell line immune response to conditioned medium from transfected RBCs

In order to evaluate the immune response elicited by GVHSV-transfected RBCs on RTG-2 cells, RTG-2 cell monolayers in 96-well plates were treated with CM from pmTFP1- or pmTFP1GVHSV-transfected RBCs. First, CM of transfected RBCs were collected at three and six days post-transfection, recovered by centrifugation (1,600 rpm), and filtered with 0.2 μm filters (Cultek). The CM was diluted 1/5 in MEM 10% FBS, and RTG-2 cell monolayers were treated with diluted CM for three days at 14°C. Finally, RTG-2 cell were stored at −80°C in lysis buffer until RNA extraction and RT-qPCR.

To evaluate the protection conferred by GVHSV-transfected RBC CM on RTG-2 cells against VHSV infection, pmTFP1- and pmTFP1GVHSV-transfected RBC CM was collected at three and six days post-transfection as described above. Then RTG-2 cell monolayers were pre-treated with the CM, diluted 1/5 and 1/125 in MEM 10% FBS, and incubated for 24 h at 14°C. Then, CM was removed and RTG-2 cell monolayers were infected with VHSV at a multiplicity of infection (MOI) of 10^−2^ in RPMI 2% FBS, for 2 h at 14°C. Medium was removed and fresh medium (RPMI 2% FBS) was added. The cells were incubated for an additional 24 h at 14°C. After that, VHSV infectivity was evaluated by means of focus forming units (FFU)/mL as previously described (9). N-VHSV antibody (2C9) was used as primary antibody. Immunofluorescence images were taken with the IN Cell Analyzer 6000 cell imaging system.

### Co-cultures of transfected RBCs with RTS11 cells

Ficoll-purified RBCs were transfected as indicated above. Transfected RBCs were co-cultured with RTS11 cells using Transwell® polyester membrane cell culture inserts (0.4 μm pore size, Costar, Corning, Sigma-Aldrich) on 24-well plates for three days at 14°C. Then, RTS11 samples were stored at −80°C in lysis buffer until RNA extraction and RT-qPCR.

### RNA extraction, cDNA synthesis, and RT-qPCR gene expression

RNA extraction, cDNA synthesis and RT-qPCR analyses were performed as previously described (8). Briefly, E.Z.N.A.® Total RNA Kit (Omega Bio-Tek, Inc., Norcross, GA) was used together with DNAse (TURBO™ DNase, Ambion, Thermo Fisher Scientific, Inc.) for RNA extraction. RNA was quantified with a NanoDrop® Spectrophotometer (Nanodrop Technologies, Wilmington, DE). After cDNA synthesis (31), RT-qPCR was performed using the ABI PRISM 7300 System (Applied Biosystems, Thermo Fisher Scientific, Inc.). Specific primers and probes are listed in Table 1. The eukaryotic 18S rRNA gene (Applied Biosystems, Thermo Fisher Scientific, Inc.) or the gene encoding EF1α were used as endogenous controls.

**Table 1:** Table of primers used in RT-qPCR.

**Gene**	**Forward primer**	**Reverse primer**	**Probe**	**Reference or accession number**
*arrb2*	GTGGAGCTGCCCTTTGTCTTA	TGAATGTGGGCGGGATATG	TGCACCCCAAACCCACAGAACTGC	NM_001171899.1
*cat*	TGCAAGACACCCCGTTCATA	TGGCGTGTACCACCCTCTCT		XM_021557350.1
*dnm2*	GTCAACAAGTCCATCAGGGATCT	CAACTCAGAATGGATGAAGTCTTTAGC		XM_021596596.1
*ef1α*	ACCCTCCTCTTGGTCGTTTC	TGATGACACCAACAGCAACA	GCTGTGCGTGACATGAGGCA	(32)
*gvhsv*	GGGCCTTCCTTCTACTGGTACTC	CGGAATCCCGTAATTTGGAAT	CTGTTGCTGCAAGGCGTCCCCT	(31)
*ifit5*	CCCTGCCCTCATCTTTCTTCT	CCCTCAATGACTCTGACAAGCA	CCAGCTTCGGCCTGTTTCTGTTCCA	AF483530.1
*ifn1*	ACCAGATGGGAGGAGATATCACA	GTCCTCAAACTCAGCATCATCTATGT	AATGCCCCAGTCCTTTTCCCAAATC	(8)
*il10*	CTGCTGGACGAAGGGATTCTA	TAAAGTCGTTGTTGTTCTGTGTTCTG	AAGTTCTATCTCGACACGGTGCTGCCC	NM_001245099.1
*il12β*	TGACAGCCAGGAATCTTGCA	GAAAGCGAATGTGTCAGTTCAAA	ACCCAACGACCAGCCTCCAAGATG	(33)
*inos*	TCAGAACCTCCTCCACAA	GTGTACTCCTGAGAGTCCTTT	GCACCGACAGCGTCTA	(33)
*jak2*	CCTGCTCTACGCCTCACAGATC	GCCAAGTCACGGTGGATGTA	CAAGGGCATGGACTACCTAGCGACCA	XM_021622657.1
*mhcI*	GACAGTCCGTCCCTCAGTGT	CTGGAAGGTTCCATCATCGT		(34)
*mhcII*	TGCCATGCTGATGTGCAG	GTCCCTCAGCCAGGTCACT	CGCCTATGACTTCTACCCCAAACAAAT	(35)
*mx1-3*	TGAAGCCCAGGATGAAATGG	TGGCAGGTCGATGAGTGTGA	ACCTCATCAGCCTAGAGATTGGCTCCCC	(36)
*nkef*	CGCTGGACTTCACCTTTGTGT	ACCTCACAACCGATCTTCCTAAAC		(8)
*nup107*	GCTGTCGCCTATTGTACGAGATG	TGAGCCTTCTTCTGAACTGAACTCT		XM_021564152.1
*pkr*	ACACCGCGTACCGATGTG	GGACGAACTGCTGCCTGAAT	CACCACCTCTGAGAGCGACACCACTTC	(8)
*prdx6*	GGACCCTGATGAGCTTGACAA	CTTATCTGGACCAATCACAAACACA		NM_001165132.2
*rab7a*	GTTGCGTGCTGGTGTTTGAC	ACTCGTCCCTCCAGCTGTCTAG	TGACCGCCCCCAACACCTTCAA	XM_021609589.1
*rora*	AGGTGGTGTTCATCAGGATGTG	CGTCGGTCCCAGCGTACTT	CGTGCCTTTGACTCTCAGAACAGCACC	XM_021608048.1
*sec13*	GCAGTGATCCAGGCACAGAA	CTGGGACTAGGATAGATGGTAGAAGTG	ATTCCACTCCTCCTCCTACCCCCACA	XM_021610740.1
*socs1*	GATTAATACCGCTGGGATTCTGTG	CTCTCCCATCGCTACACAGTTCC		(37)
*sod1*	GCCGGACCCCACTTCAAC	CATTGTCAGCTCCTGCAGTCA		(8)
*trx*	AGACTTCACAGCCTCCTGGT	ACGTCCACCTTGAGGAAAAC		(8)

### Immunofluorescence and flow cytometer assays

Transfected RBCs were fixed, permeabilized, and incubated with primary and secondary antibodies as described in Nombela et al. (9). Flow cytometry was done using a FACS Canto II (BD Biosciences, Madrid, Spain) flow cytometer. RBC populations were selected by forward scatter (FSC) and side scatter (SSC) (Figure S2). Immunofluorescence images were taken with the IN Cell Analyzer 6000 cell imaging system.

### Statistical analysis

GraphPad Prism 6 (www.graphpad.com) software was used for statistical analysis. Flowing Software (www.flowingsoftware.com) was used to analyze flow cytometry experiments.

## Results

### GVHSV expression in rainbow trout RBCs

**Figure 2:**
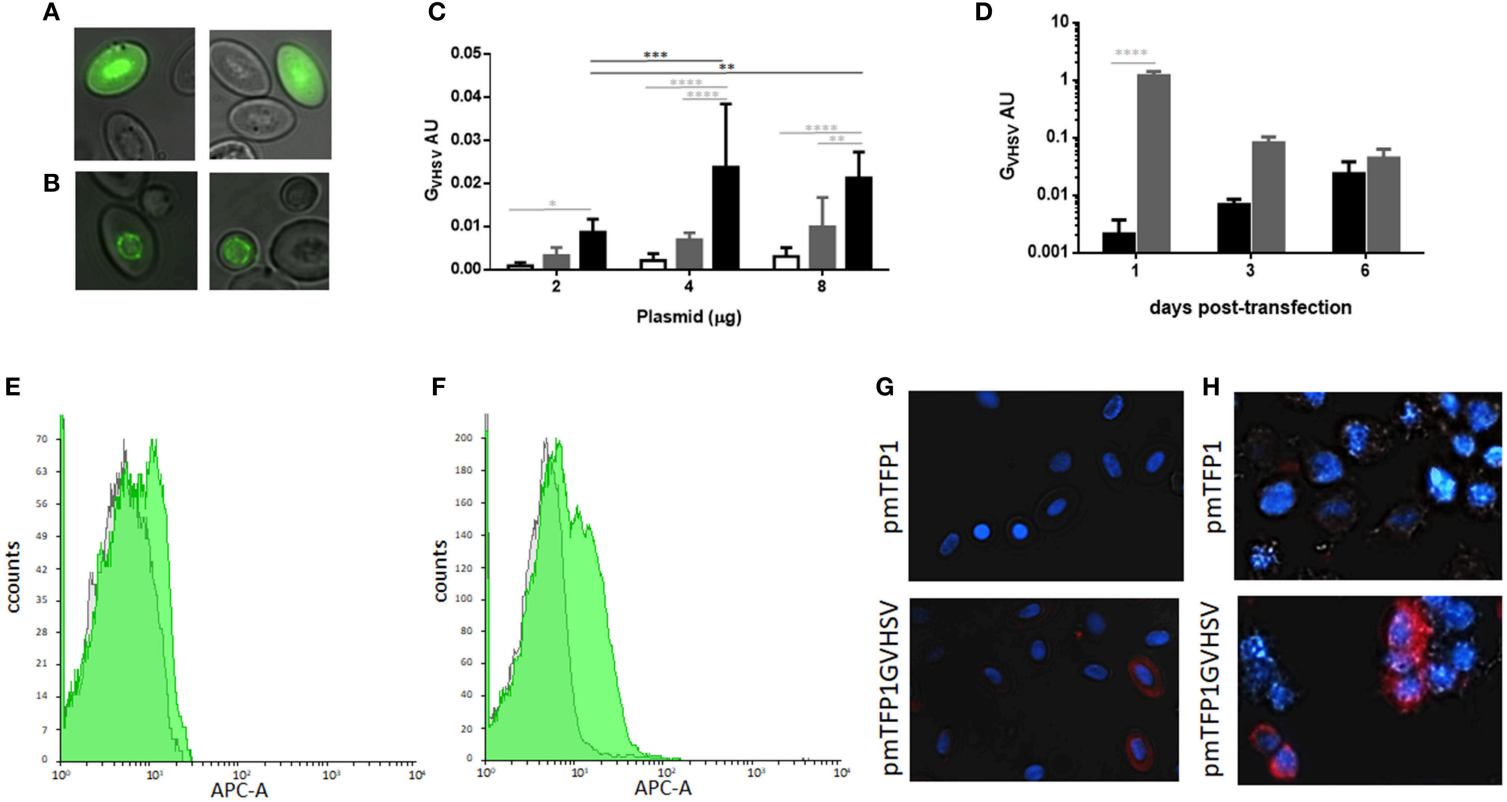
Monitoring transfection of rainbow trout red blood cells (RBCs). Fluorescent micrographs of RBCs transfected with **(A)** pmTFP1 and **(B)** pmTFP1GVHSV at six days post-transfection at 14°C, monitored by teal fluorescent protein (TFP). Fluorescent images taken with the IN Cell 6000 imaging system, augmentation 60x. **(C)** RBC transfection with 2, 4, and 8 μg of plasmid pmTFP1GVHSV was confirmed by GVHSV gene RT-qPCR, at one day (white bars), three days (gray bars) and six days (black bars) post-transfection. The eukaryotic 18S rRNA gene was used as an endogenous control. Data are displayed as mean ± SD (*n* = 3). Two-way ANOVA with Tukey's multiple comparisons test was performed between plasmid concentrations (black lines and asterisks) and times post-transfection (gray lines and asterisks). **(D)** Time course of transfected RBCs (black bars) and transfected RTS11 (gray bars) with 4 μg of pmTFP1GVHSV at one, three and six days post-transfection monitored by GVHSV RT-qPCR. The eukaryotic 18S rRNA gene was used as an endogenous control. Data are displayed as mean ± SD (*n* = 3 for RBCs and *n* = 2 for RTS11). Two-way ANOVA with Sidak's multiple comparisons test was performed between cell types at the different times post-transfection. ^*^, ^**^, ^***^, and ^****^, represent the *P* values < 0.05, < 0.01, < 0.001, and < 0.0001, respectively. Overlay flow cytometry histogram of representative GVHSV immunostaining of **(E)** RBCs and **(F)** RTS11 cells transfected with 4 μg of pmTFP1 or pmTFP1GVHSV at three days post-transfection (gray histogram, pmTFP1-transfected; green histogram, pmTFP1GVHSV-transfected). Representative immunofluorescence of GVHSV intracellular immunostaining of **(G)** RBCs and **(H)** RTS11 cells transfected with 4 μg of pmTFP1 or pmTFP1GVHSV at three days post-transfection (protein [APC, red] and nuclei [DAPI, blue]). Fluorescence images were taken with 60 × magnification.

TFP1 (Figure 2A) and GVHSV (Figure 2B) expression in transfected RBCs was monitored through fluorescent microscopy. Perinuclear expression of GVHSV was observed in pmTFP1GVHSV-transfected RBCs (Figure 2B), which is in contrast to the nuclear and cytoplasmic expression observed in pmTFP1-transfected RBCs (Figure 2A).

Time-course and dose-response assays were performed to establish the optimal conditions of pmTFP1GVHSV transfection. RBCs achieved the maximum expression of the GVHSV gene at six days post-transfection with 4 μg per 1 × 10^6^ RBCs evaluated by RT-qPCR (Figures 2C,D). These conditions were used for the following assays.

GVHSV gene expression in rainbow trout RBCs was compared with that in RTS11, another rainbow trout cell line. The RTS11 monocyte/macrophage-like cell line had higher levels of GVHSV gene expression at 24 h post-transfection (Figure 2D) than the RBCs and decreased over time. This is in contrast to pmTFP1GVHSV-transfected RBCs, which reached the maximum level of GVHSV expression at six days post-transfection. GVHSV gene expression levels were not significantly different between RBCs and RTS11 at three and six days post-transfection, although GVHSV gene expression was lower in RBCs than RTS11 at all-time points analyzed.

Also, GVHSV protein expression was lower in RBCs than RTS11 at three days post-transfection by flow cytometry (Figures 2E,G) compared to RTS11 (Figures 2F, H).

### RNA sequencing of FACS single-cell sorted GVHSV-expressing RBCs

In order to evaluate the immune response triggered by GVHSV DNA vaccine in RBCs, we analyzed the transcriptome of FACS single-cell sorted GVHSV-expressing RBCs, exclusively (Figure 1; Figure S1).

**Figure 3:**
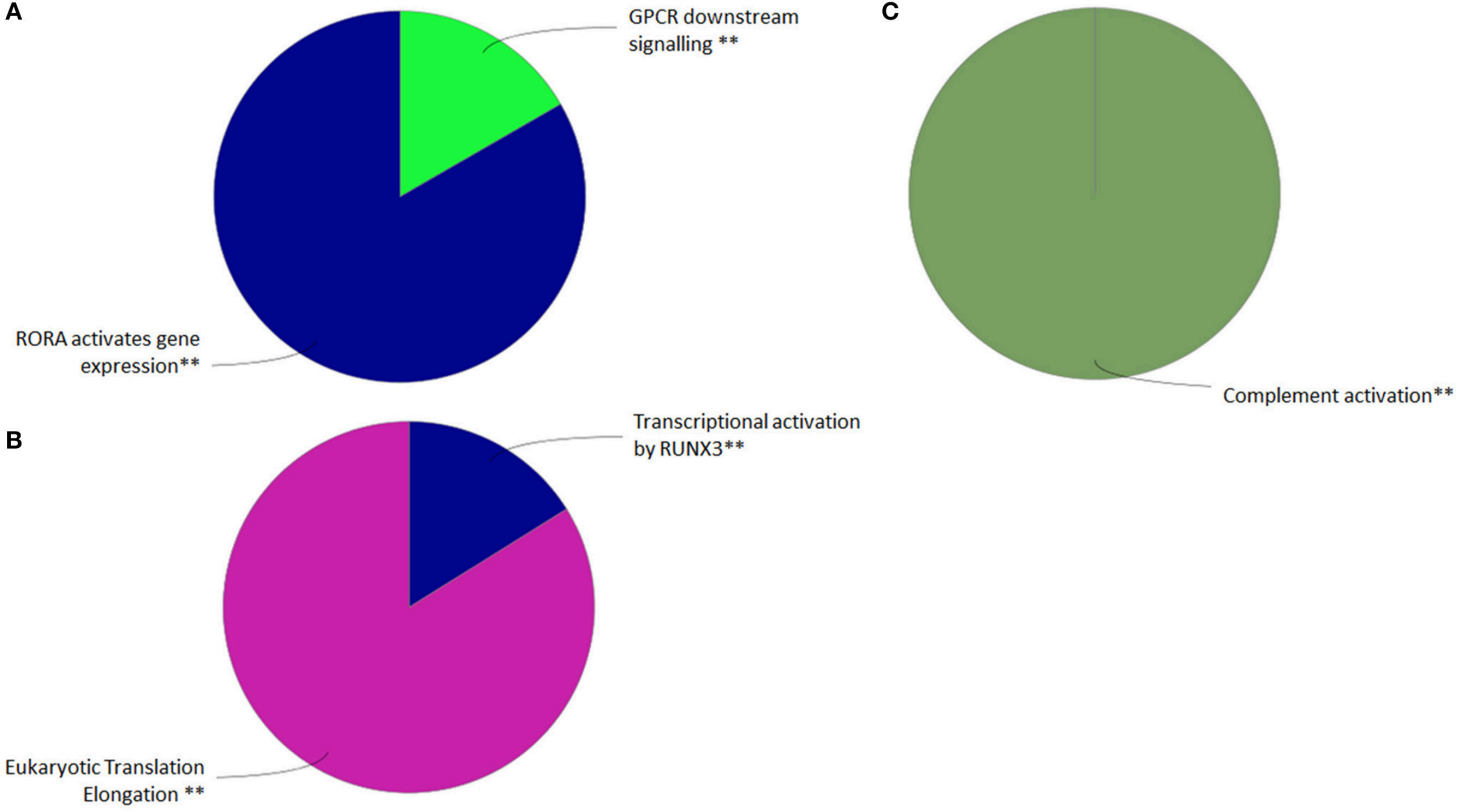
Functional pathway analysis of differentially expressed genes (DEGs) in the FACS single-cell sorted GVHSV-expressing RBC transcriptome profile. DEGs in pmTFP1GVHSV with FDR *Q* value < 0.05 and fold change *P* value < 0.05. Overrepresented GO terms were identified by the Cytoscape ClueGO app with Reactome and GO Immune Process terms. The Reactome Pathways multilevel pie chart shows **(A)** upregulated and **(B)** downregulated overrepresented terms in pmTFP1GVHSV-expressing RBCs. The GO Immune Process multilevel pie chart shows **(C)** upregulated overrepresented terms in pmTFP1GVHSV-expressing RBCs. Asterisks denote GO-term significance (^**^p < 0.01).

RNA-sequencing of FACS single-cell sorted GVHSV-expressing RBCs (compared with FACS single-cell sorted TFP1-expressing RBC) revealed 3249 DEGs (FDR < 0.05) from a total of 137,444 transcripts. Among these 3,249 DEGs, 1,786 were upregulated, and 1,463 were downregulated (Table S1). Functional pathway enrichment evaluation in FACS single-cell sorted GVHSV-expressing RBCs showed upregulation of GPCR downstream signaling and RORA-activates gene expression pathways using the Reactome Pathways Database (Figure 3A, Table S2), and the complement activation pathway using GO Immune Process Database (Figure 3C, Table S3). On the other hand, transcriptional regulation by RUNX3 and eukaryotic translation elongation pathways appeared to be downregulated using the Reactome Pathways Database (Figure 3B, Table S2).

Among all DEGs identified, we identified modulation of genes related to interferon alpha/beta signaling in antiviral mechanisms. Particularly, genes encoding suppressor of cytokine signaling 3 (*socs3*); adenosine deaminase, RNA specific (*adar*); radical s-adenosyl methionine domain-containing 2 (*rsad2*); tripartite motif-containing 25 (*trim25*); and nucleoporins such as nucleoporin 58 (*nup58*), karyopherin subunit beta 1 (*kpnb1*), and nucleoporin 210 (*nup210*) were upregulated (Table S1). Conversely, interferon regulatory factor 1 (*irf1*), interferon alpha and beta receptor subunit 1 (*ifnar1*), Janus kinase 1 (*jak1*), and major histocompatibility complex class I (*mhcI*) genes were downregulated. We also found that several DEGs related to interleukin signaling were upregulated. These included interleukin 2 receptor subunit beta (*il2rb*), *socs3*, Janus kinase 2 (*jak2*), interleukin 16 (*il16*), interleukin 15 (*il15*), interleukin 12a (*il12a*), tumor necrosis factor (*tnf*), rar-related orphan receptor α (*rora*), and interleukin 8 (*il8*) (Table S1). The complement cascade was represented by the upregulation of genes encoding complement c3 (*c3*), carboxypeptidase b2 (*cpb2*), coagulation factor II, thrombin (*f2*), and complement c1q b chain (*c1qb*) (Table S1).

### Proteome sequencing of GVHSV-transfected RBCs

**Figure 4:**
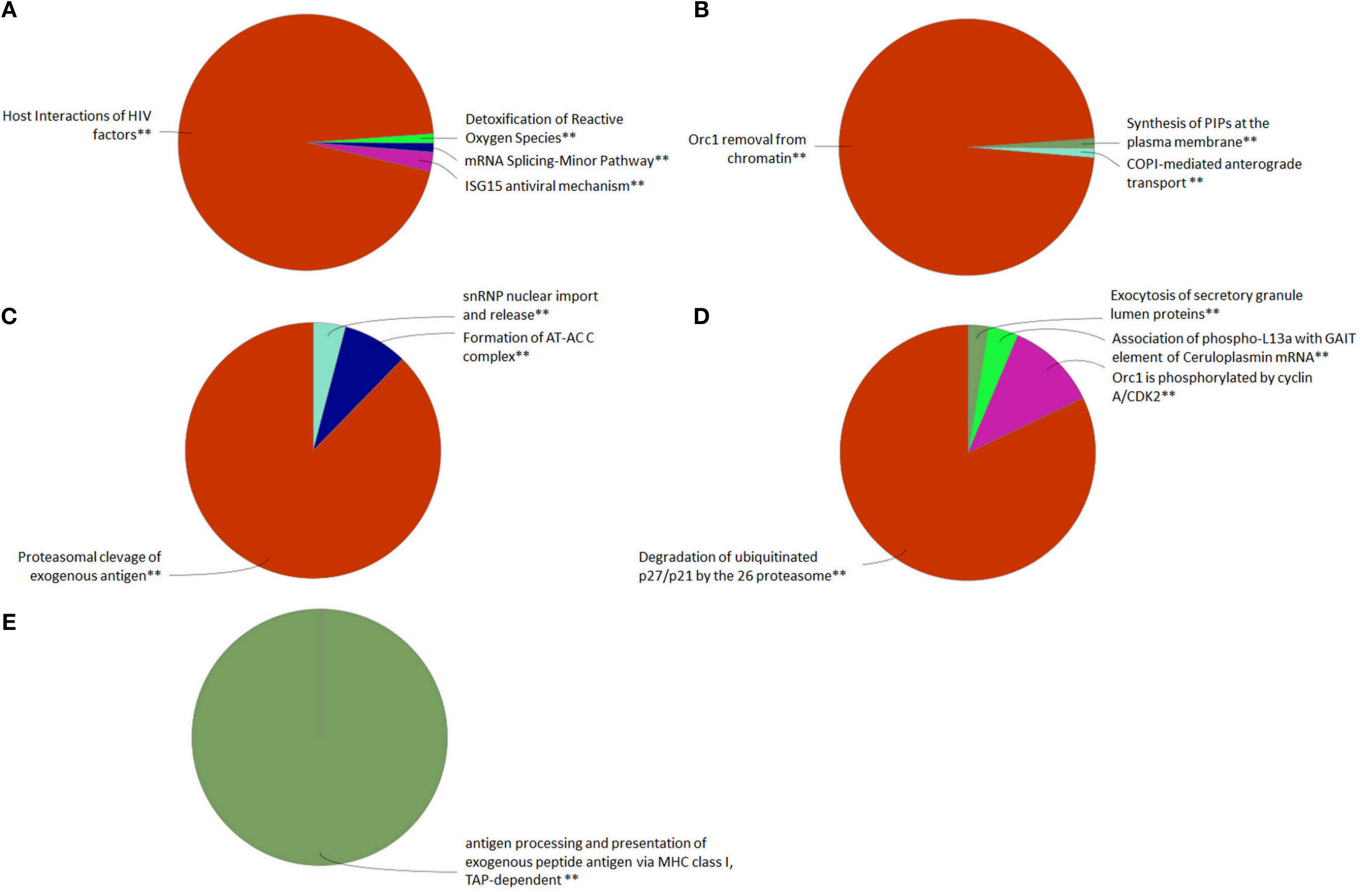
Functional pathway analysis of differentially expressed proteins (DEPs) in the GVHSV-transfected RBC proteome profile. DEPs in pmTFP1GVHSV with (−1.5 < log_2_ fold change < 1.5) and an FDR *P*-value < 0.001. Overrepresented GO terms were identified by the Cytoscape ClueGO app, with Reactome Pathways, Reactome Reactions and GO Immune Process terms. The Reactome Pathways multilevel pie chart shows **(A)** upregulated and **(B)** downregulated overrepresented terms in pmTFP1GVHSV-transfected RBCs. The Reactome Reactions multilevel pie chart shows **(C)** upregulated and **(D)** downregulated overrepresented terms in pmTFP1GVHSV-transfected RBCs. The GO Immune Process multilevel pie chart shows **(E)** upregulated overrepresented terms in pmTFP1GVHSV-transfected RBCs. Asterisks denote GO-term significance (^**^p < 0.01).

We evaluated the proteome of pmTFP1GVHSV-transfected RBCs, a sample composed of few GVHSV-expressing RBCs and mostly non–GVHSV-expressing RBCs, in order to evaluate the immune response of non–GVHSV-expressing RBCs to the signal triggered by GVHSV-expressing RBCs. Proteomic profiling identified 1,750 proteins (Table S4). After applying a filter of FDR < 0.001 and [−1.5 < Log_2_fold change (FC)> 1.5], for pmTFP1GVHSV-transfected RBCs compared to pmTFP1-transfected RBCs, 199 DEPs were identified, of which 75 were upregulated and 124 were downregulated (Table S4). ClueGO analysis using the Reactome Pathways Database revealed upregulated terms related to ISG15 antiviral mechanism, detoxification of reactive oxygen species (ROS), mRNA splicing, host interactions of HIV, CLEC7A signaling, interleukin1 family signaling, and FCERI-mediated NF-κβ factors in pmTFP1GVHSV-transfected RBCs (Figure 4A, Table S5). Conversely, downregulated terms in pmTFP1GVHSV-transfected RBCs appeared to be related to DNA replication and cell cycle regulation, Orc1 removal from chromatin, synthesis of PIPs at the plasma membrane, and COPI-mediated anterograde transport (Figure 4B, Table S5). ClueGO analysis using Reactome Reactions Database showed upregulated terms related to proteosomal cleavage of exogenous antigen, snRNP nuclear import and release, and formation of the AT-AC C complex in pmTFP1GVHSV-transfected RBCs (Figure 4C, Table S6). On the other hand, downregulated terms included degradation of ubiquitinated p27/p21 by the 26S proteasome, Orc1 phosphorylation by cyclin A/CDK2, association of phospo-L13a with GAIT element of ceruloplasmin mRNA and exocytosis of secretory granulate lumen proteins (Figure 4D, Table S6). ClueGO analysis using the GO Immune Process Database identified antigen processing and presentation of exogenous peptide terms in pmTFP1GVHSV-transfected RBCs (Figure 4E, Table S7).

Among the most upregulated DEPs in pmTFP1GVHSV-transfected RBCs (Table S4), we identified proteins related to: (i) the nuclear pore complex, such as importin-8 (IPO8), nuclear pore complex protein Nup107 (NUP107), and translocated promoter region nuclear basket protein (TPR); (ii) inhibitor of nuclear factor kappa-B kinase subunit alpha (CHUK), and (iii) protection against oxidative stress such as thioredoxin (TRX), peroxiredoxin 4 (PRDX4), superoxide dismutase 1 (SOD1), and thioredoxin like 1 (TXNL1).

### Validation of upregulated pathways by RT-qPCR

**Figure 5:**
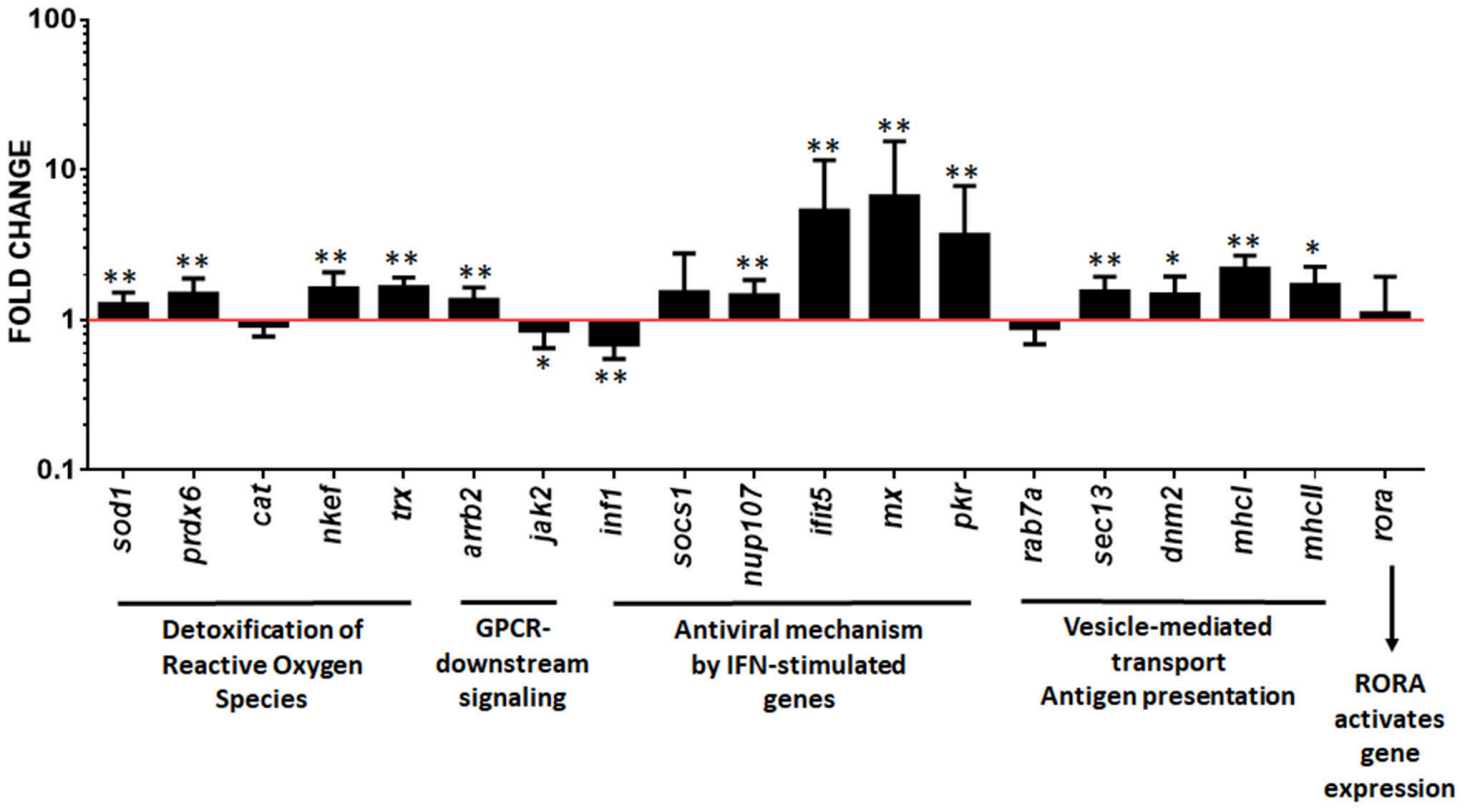
Gene expression evaluation to validate functional pathway analysis of differentially expressed genes (DEGs) and differentially expressed proteins (DEPs) in GVHSV-transfected RBCs transcriptome and proteome profiles, respectively. RBCs were transfected with pmTFP1 and pmTFP1GVHSV plasmids for 6 days at 14°C. Afterwards, gene expression was evaluated by RT-qPCR. Data are displayed as mean ± SD (*n* = 5). The EF1α gene was used as an endogenous control. The Wilcoxon test was performed between pmTFP1GVHSV- and pmTFP1-transfected RBCs (control, red line). ^*^and ^**^represent *P-*values < 0.05 and < 0.01, respectively.

Upregulated pathways in pmTFP1GVHSV-transfected RBCs were validated via RT-qPCR analysis. Genes and proteins were selected from each pathway for validation. For the GPCR-downstream signaling term (which was upregulated in the RNA sequencing results of FACS single-cell sorted GVHSV-expressing RBCs), we measured gene expression levels of the arrestin beta 2 (*arrb2*) gene, which was significantly upregulated, and the Janus kinase 2 (*jak2*) gene, which was significantly downregulated in our RT-qPCR results in contrast to RNA sequencing results (Figure 5). The *rora* gene, a representative gene of RORA-activates gene expression pathway, an overrepresented term in RNA sequencing results of FACS single-cell sorted GVHSV-expressing RBCs, was significantly upregulated in RT-qPCR results (Figure 5). For the detoxification of reactive oxygen species pathway (found to be upregulated in the proteome analysis of pmTFP1GVHSV-transfected RBCs), the superoxide dismutase 1 (*sod1*), peroxiredoxin 6 (*prdx6*), natural killer enhancing factor (*nkef*), and thioredoxin (*trx*) genes were significantly upregulated (Figure 5). For the ISG15 antiviral mechanism pathway (which was upregulated in the proteome analysis of pmTFP1GVHSV-transfected RBCs), the nucleoporin 107 (*nup107*), interferon-induced protein with tetratricopeptide repeats 5 (*ifit5*), interferon-inducible Mx (*mx*), and interferon-inducible RNA-dependent protein kinase (*pkr*) genes were significantly upregulated. However, the interferon type 1 (*ifn1*) gene was significantly downregulated (Figure 5). For the antigen presentation of exogenous peptide pathways (upregulated in proteome analysis of pmTFP1GVHSV-transfected RBCs), the major histocompatibility complex class I (*mhcI*) and II (*mhcII*), SEC13 homolog-nuclear pore and COPII coat complex component (*sec13*), and dynamin 2 (*dnm2*) genes were significantly upregulated, but the RAB7A-member RAS oncogene family (*rab7a*) appeared to be slightly downregulated in contrast to proteomic results (Figure 5).

**Figure 6:**
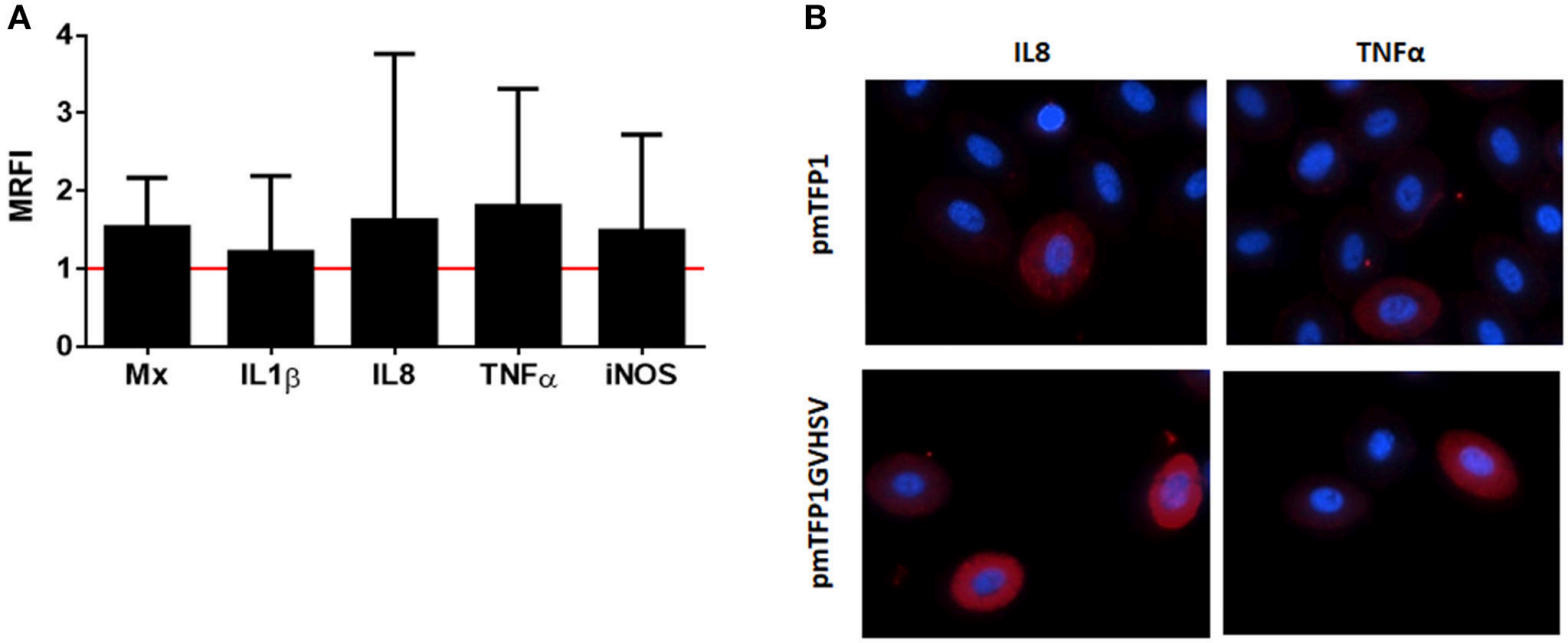
Immune proteins expression in GVHSV-transfected RBCs. **(A)** Immune protein expression measured by flow cytometry and calculated by the formula MRFI (mean relative fluorescence intensity) = fluorescence in pmTFP1GVHSV- transfected RBCs MRFI (mean relative fluorescence intensity) = fluorescence in pmTFP1GVHSV- transfected RBCs /fluorescence in pmTFP1-transfected RBCs. **(B)** Representative immunofluorescence of pmTFP1- and pmTFP1GVHSV-transfected RBCs (protein [APC, red] and nuclei [DAPI, blue]). Fluorescence images were taken with 60 × magnification.

However, at a protein level, we confirmed the upregulation of interferon inducible Mx protein, and interleukins interleukin 1 beta (IL1β), interleukin 8 (IL8), and tumor necrosis factor alpha (TNFα) measured by flow cytometry (Figures 6A, B).

### Protection conferred by pmtfp1gvhsv-transfected RBC CM on RTG-2 cells

**Figure 7:**
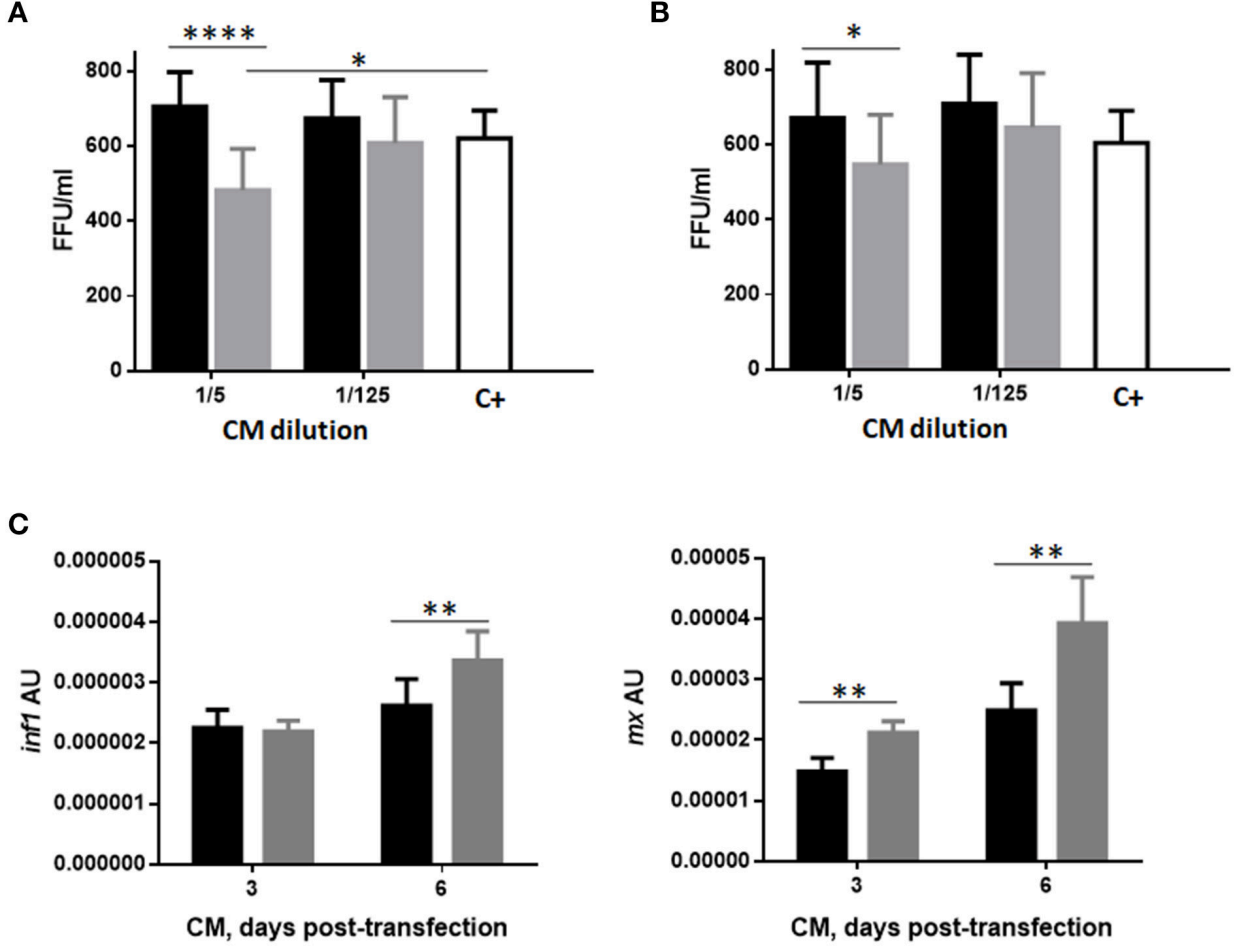
Protection conferred by conditioned medium (CM) from GVHSV-transfected RBCs against VHSV infection in RTG-2 cells. RTG-2 cells pretreated with CM from RBCs transfected with pmTFP1 (black bars) and pmTFP1GVHSV (gray bars) plasmids (diluted 1/5 and 1/125 in MEM 10% FBS), at three **(A)** and six **(B)** days post-transfection. Cells were then infected with VHSV (MOI 1 × 10^−2^) for 24h at 14°C. The positive control is non-pretreated RTG-2 cells infected with VHSV. VHSV infectivity was evaluated by means of focus forming units (FFU)/mL. FFUs were stained with 2C9 antibody against the N protein of VHSV. Data are displayed as mean ± SD (*n* = 3). The Mann Whitney test was performed between treatments at each dilution. **(C)** *ifn1* and *mx* gene expression in RTG-2 cells quantified by RT-qPCR after treatment with CM from pmTFP1- (black bars) and pmTFP1GVHSV- (gray bars) transfected RBCs (diluted 1/5 in MEM 10% FBS) at three and six days post-transfection. The eukaryotic 18S rRNA gene was used as an endogenous control. Data are displayed as mean ± SD (*n* = 3). The Mann Whitney test was performed between conditions. ^*^, ^**^, and ^****^, represent *P-*values < 0.05, < 0.01, and < 0.0001, respectively.

In order to evaluate the capacity of RBCs to propagate the immune response elicited by GVHSV to other cell types, we measured the protection conferred by pmTFP1GVHSV-transfected RBC CM to RTG-2 cells against VHSV infection. Treatment of RTG-2 cells with three or six days pmTFP1GVHSV-transfected RBC CM significantly decreased VHSV compared with pmTFP1-transfected RBC CM (Figures 7A,B, for three and six days transfected RBC CM, respectively).

To determine whether this protection was due to the stimulation of type 1 interferon signaling in RTG-2 cells, we evaluated the expression of *ifn1* and interferon-inducible *mx* genes in RTG-2 cells incubated with pmTFP1- or pmTFP1GVHSV-transfected RBC CM (Figure 7C). We observed significant upregulation of *ifn1* gene expression in RTG-2 cells treated with pmTFP1GVHSV-transfected RBC CM at six days post-transfection and of *mx* gene expression at three and six days post-transfection.

### Crosstalk between transfected RBCs and RTS11

**Figure 8:**
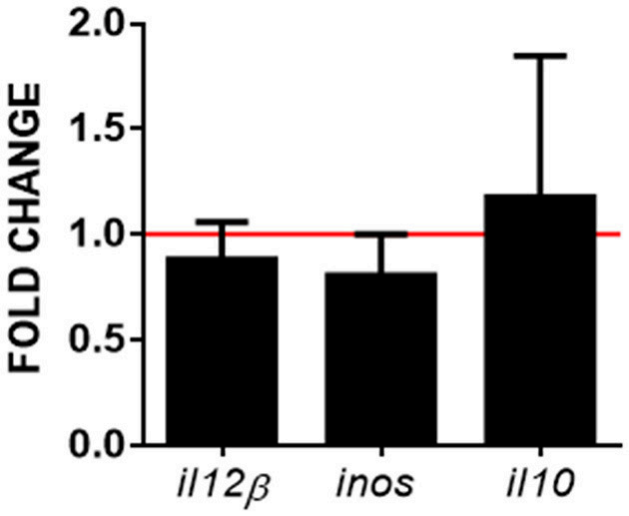
M1 and M2 macrophage markers in RTS11 cells co-cultured with GVHSV-transfected RBCs. Rainbow trout RBCs transfected with pmTFP1 or pmTFP1GVHSV for three days at 14°C. Afterwards, transfected RBCs were co-cultured with the RTS-11 cell line at 14°C for three days. Then, the *il12*β, *inos*, and *il10* gene expression profiles were quantified by RT-qPCR. Gene expression was normalized against eukaryotic 18S rRNA and compared to control cells (RTS11 co-cultured with pmTFP1-transfected RBCs, red line) (fold-change). Data represent the mean ± SD (*n* = 6). A Wilcoxon test was performed between both conditions.

In order to evaluate whether pmTFP1GVHSV-transfected RBC CM could induce monocyte/macrophage differentiation, we co-incubated transfected RBCs with RTS11, a monocyte/macrophage-like cell line. At three days post-transfection, pmTFP1-and pmTFP1GVHSV-transfected RBCs were co-cultured with RTS11 cells for three days. Using RT-qPCR, we observed slight, but not significant, upregulation of RTS11 differentiation markers The slight upregulation of the interleukin 10 (*il10*) gene, a marker of M2 macrophages (38) was accompanied by a slight downregulation of interleukin 12 subunit beta (*il12*β) and inducible nitric oxide synthase (*inos*), which are markers of M1 macrophages (Figure 8).

## Discussion

Recent studies have implicated nucleated RBCs in the immune response to viral infections in fish, as these cells are able to actively transcribe and transduce signaling molecules in response to viral attack (2). Moreover, although DNA vaccines are mainly delivered via intramuscular injection, non-nucleated RBCs are thought to be promising drug and vaccine carriers (39, 40, 41, 42) by eliciting humoral immune responses comparable or superior to those obtained via the subcutaneous vaccination route (39). In this study our aim was to elucidate the role of nucleated RBCs in the immune response to DNA vaccines in order to explore their usefulness in improving immune response to DNA vaccines in fish.

As far as we know, this is the first report of fish nucleated RBCs expressing the antigen encoded by a DNA vaccine *in vitro*. Besides, we have not found any report showing nucleated RBCs expressing the protein encoded by a DNA vaccine *in vivo*. GVHSV-transfected RBCs showed a characteristic perinuclear expression of GVHSV protein that appeared in the perinuclear region of stressed RBCs or on the membrane of shape-shifted RBCs (shRBC) (33). shRBCs are small, round cells with a thin membrane derived from RBCs subjected to stressful conditions. Translation of the GVHSV gene into protein and translocation to the cell membrane induces RBC transformation into stressed RBC and shRBC (33). Like RBCs, shRBCs have been shown to participate in roles related to immune response and homeostasis (33).

Transcriptome profiling of single-cell sorted GVHSV-expressing RBCs demonstrated the capacity of RBCs to modulate the expression of genes related to innate and adaptive immune responses in response to the DNA vaccine. Among the pathways upregulated in single-cell sorted GVHSV-expressing RBCs, GPCR-downstream signaling and RORA activates gene expression were the most represented. GPCRs constitute a large protein family of receptors that detect molecules outside the cell and activate internal signal transduction pathways (43). GPCRs are known to play a role in inflammation and are widely targeted in drug discovery (44). Immune cells such as monocytes and macrophages express a large number of GPCRs for classic chemoattractants and chemokines (44, 45). These receptors are critical for phagocyte migration and accumulation at sites of inflammation, where these cells can intensify inflammation or contribute to its regulation (44, 45). Among the molecules found downstream of GPCRs, β-arrestins are known to be key regulators of GPCR signaling through interaction with the Iκ*βα* component of the NF-κβ signaling complex (44, 45). Consistent with these effects on signaling, knockdown of ARRB2 has been described to enhance the expression of the NF-κβ target proteins IL6 and IL8 in response to proinflammatory stimulus (46). After RNA sequencing and RT-qPCR, we observed that the *arrb2* gene was significantly upregulated in GVHSV-transfected RBCs. We also observed that the IL1β, IL8, and TNFα proteins were slightly upregulated in response to GVHSV transfection. However, the *il8* and *tnf*α genes appeared to be highly upregulated in single-cell sorted GVHSV-expressing RBCs, and the *il6* gene appeared to be highly downregulated. In this regard, it would be interesting to study the implication of β-arrestins in RBCs regulation of the proinflammatory response. Considering the RORA activates gene expression pathway (upregulated in single-cell sorted GVHSV-expressing RBCs), it has been described that RORA is a nuclear receptor highly expressed in Th17 cells that regulates differentiation of Th17 cells (47). Moreover, RORA depletion has been reported to attenuate cytokine production (48) and has thus demonstrated its involvement in inflammatory responses. The signaling paradigms of GPCRs and RORA in inflammatory regulation and immune cell differentiation in nucleated RBCs remain to be studied and are part of our ongoing research.

Interestingly, GVHSV-transfected RBC CM could switch RTS11 monocyte/macrophage differentiation markers, upregulating the *il10* gene [a marker of M2 macrophages (38)] and downregulating the *il12*β and *inos* genes [markers of M1 macrophages (38)]. M1 macrophages are known to be activated by LPS and IFNγ and secrete high levels of IL12 and low levels of IL10. On the other hand, M2 macrophages are alternatively activated by certain cytokines such as IL4, IL10, or IL13 and produce high levels of IL10 and TGFβ and low levels of IL12. Their function is implicated in constructive processes like wound healing and tissue repair and in anti-inflammatory responses. The cytokines or molecules secreted by GVHSV-transfected RBCs responsible for inducing M2 macrophages markers in RTS11 are unknown and should be further investigated.

Genes related to the complement pathway were also overrepresented in single-cell sorted GVHSV-expressing RBCs. The complement system is an essential part of the innate immune response and acts as a connection between innate and acquired immunity [reviewed in Nesargikar et al. (49)]. The complement system is known to mediate responses to inflammatory triggers, leading to clearance of foreign cells through pathogen recognition, opsonization and lysis (50). On the other hand, genes and proteins related to proteosomal cleavage of exogenous antigen and antigen presentation of exogenous peptides were also upregulated in GVHSV-transfected RBCs (via MHCI or MHCII pathways), indicating that RBCs may have the capacity to present DNA vaccine antigens as has been recently reported (26). Further research is needed to determine whether RBCs are functionally capable of inducing T cell activation upon antigen presentation on their membrane.

Proteomic sequencing of GVHSV-transfected RBCs, a sample containing both few GVHSV-expressing RBCs and non–GVHSV-expressing RBCs, revealed the upregulation of ISG15 antiviral mechanisms. ISG15 is a member of the ubiquitin-like (UBL) family. ISG15 conjugates with several target proteins in a process termed ISGylation. Hundreds of target proteins have been identified in ISGylation. Among them, several proteins that are part of antiviral signaling pathways, such as Mx1 or PKR, have been identified as targets for ISGylation (51). Upregulation of the ISG15 antiviral mechanism pathway was confirmed by gene expression analysis of effector molecules within the pathway such as *mx, pkr*, and *ifit5*. The Mx, PKR, and IFIT proteins are known interferon-inducible antiviral effectors (51). Surface expression of the GVHSV protein by GVHSV-transfected cells has been reported to be a major mechanism of interferon induction (52), and VHSV infection and GVHSV vaccination have been demonstrated to induce ISGs such as *isg15* (53) and *mx* (16, 17). However, *ifn1* appeared to be downregulated in GVHSV-transfected RBCs by RT-qPCR and in single-cell sorted GVHSV-expressing RBCs by RNA sequencing. Also, RNA sequencing data showed the downregulation of genes related to interferon alpha/beta signaling such as *irf1, ifnar1*, and *jak1*. However, VHSV has been reported to induce *ifn1* downregulation in rainbow trout RBCs (8). These differences between *ifn1* and ISG gene expression could be due to the effort of the immune system to maintain homeostasis or to the differential regulation of these genes. Alternatively, it has been reported that Mx induction could be independent of interferon in HIV infection (54, 55). In addition, infectious salmon anemia (ISA) virus could trigger *mx* and *isg15* stimulation but not *ifn1* gene expression, suggesting ISG stimulation independent of interferon (56). Despite the fact that *ifn1* gene expression was downregulated in GVHSV-transfected RBCs, the IFN protein, which could be differently expressed to *inf* gene, or other cytokines or molecules secreted by GVHSV-transfected RBCs, were able to stimulate *ifn1* and *mx* gene expression as well as induce protection against VHSV infection in RTG-2 cells.

Another interesting pathway identified during the proteomic profiling of GVHSV-transfected RBCs was detoxification of reactive oxygen species (ROS). Gene expression of antioxidant enzymes such as *sod1, nkef* , *prdx6*, and *trx* appeared to be upregulated in GVHSV-transfected RBCs. This mechanism has been reported in rainbow trout RBCs exposed to VHSV (8), where protective antioxidant enzymes were implicated in the response of RBCs to the induction of ROS after viral exposure. However, in this study, it is important to note that the antigen GVHSV encoded by this DNA vaccine is able to induce ROS signaling and homeostasis.

In summary, rainbow trout nucleated RBCs were able to induce immune responses to the DNA vaccine and send signals to neighboring cells or other cell types. This reveals a new approach to explore the function of RBCs in the complex teleost immune system and could prompt development in the field of vaccination with RBCs as targets or carrier cells for immunostimulation. Future studies will be focused on the molecules of interest produced by GVHSV-expressing RBCs in order to identify future vaccination targets.

## Ethics statement

Experimental protocols and methods of the experimental animals were reviewed and approved by the Animal Welfare Body and the Research Ethics Committee at the University Miguel Hernandez (approval number 2014.205.E.OEP; 2016.221.E.OEP) and by the competent authority of the Regional Ministry of Presidency and Agriculture, Fisheries, Food and Water supply (approval number 2014/VSC/PEA/00205). Besides, all methods were carried out in accordance with the Spanish Royal Decree RD 53/2013 and EU Directive 2010/63/EU for the protection of animals used for research experimentation and other scientific purposes.

## Author contributions

SP-M performed experiments, analyzed data, and wrote the manuscript. IN and VC performed experiments. SC and MM performed proteomic sequencing. LM provided valuable antibodies for the experiments. MO-V conceived ideas, oversaw the research, and co-wrote the manuscript. VC and JC contributed to the preparation of the manuscript.

### Conflict of interest statement

The authors declare that the research was conducted in the absence of any commercial or financial relationships that could be construed as a potential conflict of interest.
