# Supplementary figures and images for: Rainbow Trout Erythrocytes ex vivo Transfection With a DNA Vaccine Encoding VHSV Glycoprotein G Induces an Antiviral Immune Response
Source: Front Immunol. 2018 Oct 29;9:2477. doi: 10.3389/fimmu.2018.02477 (PMC6220650; doi:10.3389/fimmu.2018.02477)

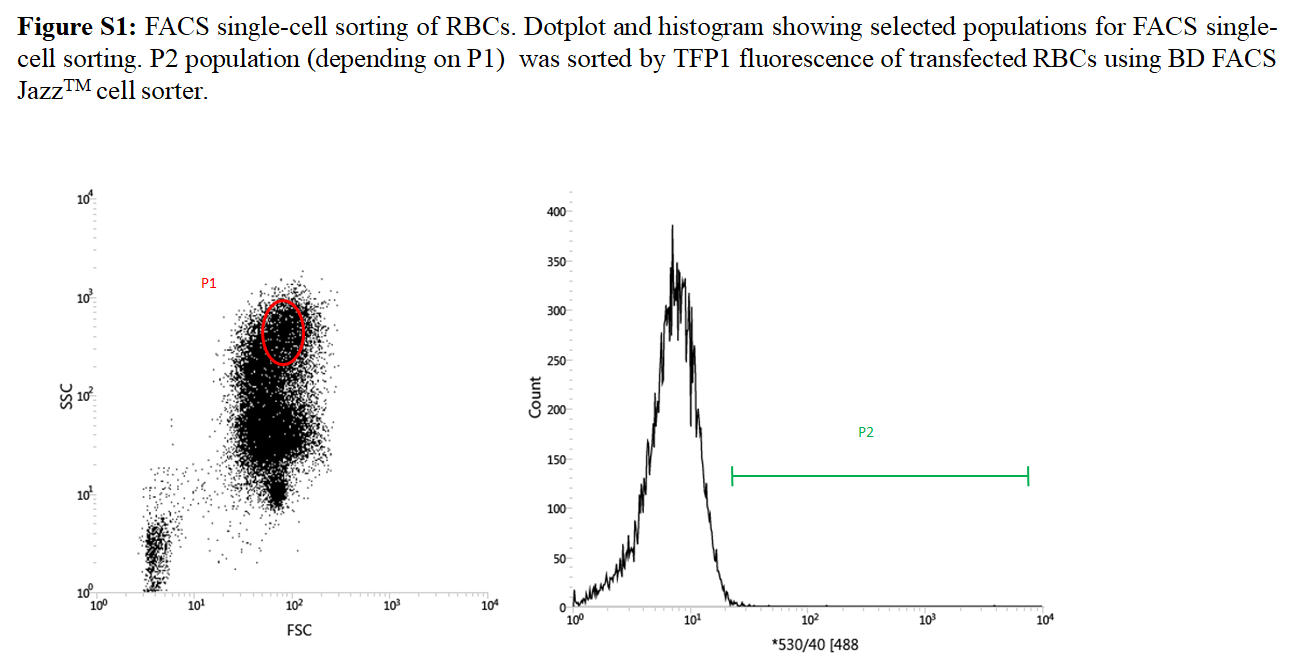

Supplement: Supplementary file 8 [file Image_1.TIF]

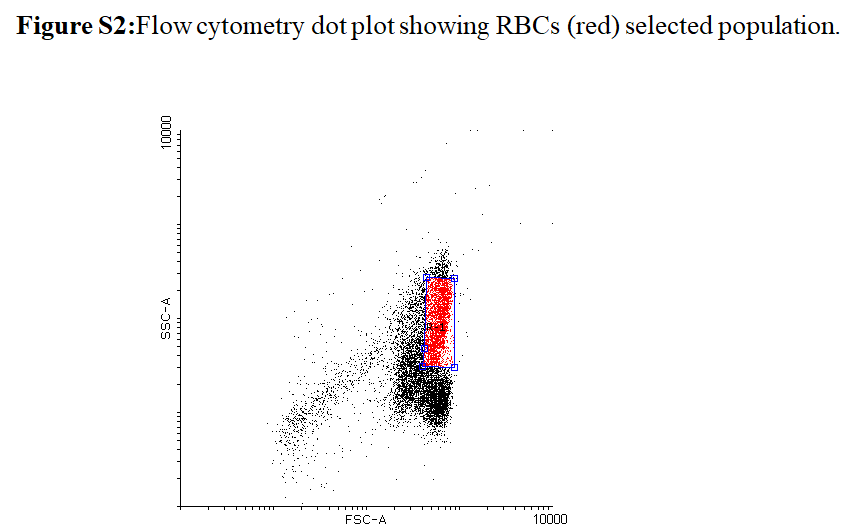

Supplement: Supplementary file 9 [file Image_2.TIF]
